# Social Anxiety Disorder in The Visually Impaired Versus Healthy Control: Saudi Arabian Samples

**DOI:** 10.1192/j.eurpsy.2025.1009

**Published:** 2025-08-26

**Authors:** S. Alsubaie

**Affiliations:** 1 Psychiatry, AFHSR, Khamees Mushait, Saudi Arabia

## Abstract

**Introduction:**

While the relationship between social anxiety disorder SAD and various sociodemographic factors has been explored, there is a notable gap in research examining the prevalence of SAD in visually impaired individuals. Vision loss could influence social interactions and thus may alter the typical presentation or severity of social anxiety.

**Objectives:**

This study aims to compare self-esteem and social anxiety disorder (SAD) levels between visually impaired and sighted individuals and to explore the relationship between social anxiety and various sociodemographic factors.

**Methods:**

A case-control study was conducted from March to June 2017 in Riyadh, Saudi Arabia, involving 62 participants (24 visually impaired and 38 sighted). Participants completed a demographic form, the Liebowitz Social Anxiety Scale (LSAS), and the Rosenberg Self-Esteem Scale (RSES). Data analysis included descriptive statistics, t-tests, and ANOVA to compare psychological outcomes between groups.

**Results:**

The mean age of visually impaired participants was significantly higher than that of sighted participants (24±2.8 vs. 22.4±2.2 years, p=0.013). No significant differences were observed between the two groups in terms of gender, marital status, or education level. The RSES scores indicated no significant difference in self-esteem between visually impaired and sighted individuals (18.13±2.66 vs. 17.42±2.04, p=0.244). Similarly, LSAS scores did not significantly differ between the two groups (32.63±24.19 vs. 36.68±22.68, p=0.506).

**Conclusions:**

The findings suggest that visually impaired individuals do not have significantly different levels of self-esteem or social anxiety compared to their sighted peers, indicating that visual impairment may not directly contribute to lower self-esteem or higher social anxiety. Future research should involve larger, more diverse samples and longitudinal studies to further explore these relationships.

**Disclosure of Interest:**

None Declared

